# Posterior Reversible Encephalopathy Syndrome and Eclampsia

**DOI:** 10.21980/J8H64T

**Published:** 2025-07-31

**Authors:** Kristina Jacomino, Kevin Tomecsek, Andrew Little, Mary Mclean

**Affiliations:** * AdventHealth East Orlando, Department of Emergency Medicine, Orlando, FL

## Abstract

**Audience:**

Emergency medicine residents, fellows, and recent graduates. Emergency medicine-bound senior medical students.

**Introduction:**

Posterior reversible encephalopathy syndrome (PRES) is an illness in which a person can present with acutely altered mentation, drowsiness or sometimes stupor, visual impairment, seizures (focal or general tonic-clonic), and sudden or constant, non-localized headaches.^1^ Patients at risk for developing PRES include those with underlying hypertension, preeclampsia, kidney disease, liver disease, exposure to cytotoxic medications or immunosuppressants, autoimmune disorders or sepsis. As a syndrome, PRES has gone underdiagnosed given its broad symptomatology. While it appears to affect people of all ages, it is more commonly found in middle-aged females. The underlying cause for PRES remains unclear, but some proposed mechanisms center on the dysregulation of cerebral autoregulation, the brain’s ability to maintain constant cerebral blood flow over a range of blood pressures via the constriction or dilation of the cerebral blood vessels.^2^ The treatment for PRES includes management of hypertension as well as diagnosing and treating the underlying etiology. This disease process needs to be recognized early by the emergency provider to reduce mortality.

Eclampsia and other hypertensive disorders in women affect as many as 10% of all pregnancies worldwide and are responsible for approximately 10% of all maternal deaths in the United States.^3^ Eclampsia is defined as new onset seizures in a woman with a history of preeclampsia who is between 20 weeks gestation and within four weeks postpartum.^4^ As an emergency medicine provider, it is imperative to be able to manage and treat a patient with eclampsia to decrease mortality and morbidity of the mother and fetus. Management of eclampsia includes treatment for seizures using magnesium sulfate, treatment for hypertension, and emergent obstetrics consult for possible delivery of the fetus.^4^

**Educational Objectives:**

At the end of this oral boards session, examinees will be able to: 1) demonstrate familiarity with the structured interview oral board format and case play; 2) recognize the history and exam features concerning for PRES and eclampsia; 3) order appropriate diagnostic workup for postpartum and hypertensive emergencies including eclampsia and PRES; 4) understand treatment options for the management of eclampsia (intravenous [IV] magnesium sulfate, IV antihypertensive therapy, and emergent consultation with an obstetrician [OB/GYN]); 5) understand threshold for taking control of airway in patients with eclampsia; 6) understand indications for ordering brain imaging in patients with eclampsia and altered mental status; and 7) demonstrate effective communication with treatment team/family members as well as correct disposition of the patient to a higher level of care (intensive care unit [ICU]).

**Educational Methods:**

An oral board exam-style structured interview (SI) case format was used. This allowed the learner to delve into the case in a methodical way while laying out their thought processes to better assess their medical knowledge. The case was administered as part of a multi-institution virtual Mock Oral Boards Day. Case material and instructions were distributed a week ahead of time to faculty examiners for preparation.

**Research Methods:**

Both learners and instructors provided written feedback after case administration. Participants gave feedback on the overall difficulty and quality of the case and provided narrative feedback on the case materials. Participants also rated the perceived effectiveness level for assessing examinees on the eight stages of patient interaction.

**Results:**

Of 49 examinees and six faculty examiners, 42 and four gave feedback on the case, respectively, for an overall 84% response rate. On a Likert scale from 1 (least effective) to 5 (most effective), learners rated the case at a mean 3.9 and faculty rated the case at a mean 4.3 across the eight structured stages of patient interaction. Case difficulty was rated *intermediate/advanced* overall. On a Likert scale from 1 (lowest quality) to 5 (highest quality), learners gave a mean rating of 3.9 and faculty gave a mean rating of 4.0. Narrative comments recommended better clarifying the history, adding a point-of-care glucose to the workup, and allowing varied magnesium sulfate dosages within the recommended range for eclampsia, and these recommendations were used to improve the case. Outside of medical knowledge aspects of this feedback, there were also comments about the structured interview format being confusing in general.

**Discussion:**

The educational content was found to be effective, high-quality, and intermediate-to-advanced in difficulty by both learners and faculty. From this implementation, we discovered that learners need more instruction on magnesium dosing in severe eclampsia, and also on the likelihood of concurrent PRES. Outside of the main medical knowledge take-away lessons, we have also gained insight about lack of familiarity with the structured interview format on the part of both examinees (learners) and examiners (faculty).

**Topics:**

Posterior reversible encephalopathy syndrome, eclampsia, preeclampsia, seizures, end-organ damage, hypertensive emergency, altered mental status, neurologic emergency, obstetric emergency, peripartum emergency, postpartum emergency.

## USER GUIDE

**Table:** 

List of Resources:	
Abstract	34
User Guide	36
For Examiner Only	39
Oral Boards Assessment	45
Clinical Decision-Making Task Sheet	47
Stimulus	48
Debriefing and Evaluation Pearls	56

**Learner Audience:**

Advanced Medical Students, Interns, Junior Residents, Senior Residents, Recent Residency Graduates

**Time Required for Implementation:**

Case: 15 min by American Board of Emergency Medicine (ABEM) standard

Debriefing: five minutes per case

**Learners per instructor:**

One learner per instructor

**Topics:**

Posterior reversible encephalopathy syndrome, eclampsia, preeclampsia, seizures, end-organ damage, hypertensive emergency, altered mental status, neurologic emergency, obstetric emergency, peripartum emergency, postpartum emergency.

**Objectives:**

By the end of this structured interview case, learners will be able to:

1. Demonstrate familiarity with the structured interview oral board format and case play.
2. Recognize history and exam features concerning for PRES and eclampsia.
3. Order appropriate diagnostic workup for postpartum and hypertensive emergencies including eclampsia and PRES.
4. Understand medication orders for management of eclampsia.
5. Understand threshold for taking control of airway in patients with eclampsia.
6. Understand indications for ordering brain imaging in patients with eclampsia and altered mental status.
7. Demonstrate effective communication with treatment team/family members as well as correct disposition of the patient to a higher level of care (ICU).

### Linked objectives, methods and results

The structured interview (SI) format was chosen to implement the above learning objectives because it allows the learner to think through an essential, cornerstone-of-emergency medicine (EM) case in a methodical way while simultaneously allowing the learner to gain experience with the new oral boards SI format (Objective 1). The learners need to verbalize their thought process as they work through differentials for this new onset seizure patient (Objective 2) as well as their rationale for ordering their specific work up (Objectives 3 and 6). They are then challenged to list out their next-steps of treatment (Objective 4) including emergent airway management (Objective 5). The final prompt encourages the learners to synthesize their interventions and disposition the patient appropriately (Objective 7).

### Recommended pre-reading for instructor

- Oral Exam. American Board of Emergency Medicine. Accessed 13 June 2024. https://www.abem.org/public/become-certified/oral-exam
- Chudnofsky C. ABEM | Virtual Oral Exam Structured Interview Case. YouTube. May 14, 2024. Accessed June 13, 2024. https://www.youtube.com/watch?v=6jSxti72WXM
- Young JS. Maternal emergencies after 20 weeks of pregnancy and in the peripartum period. In: Tintinalli JE, Ma O, Yealy DM, et al. eds. *Tintinalli's Emergency Medicine: A Comprehensive Study Guide*, 9e. McGraw-Hill Education; 2020.
- Baumann BM. Systemic hypertension. In: Tintinalli JE, Ma O, Yealy DM, et al. eds. *Tintinalli's Emergency Medicine: A Comprehensive Study Guide*, 9e. McGraw-Hill Education; 2020.
- Meloy PG, Henn MC, Rutz D, Bhambri A. Eclampsia. *J Educ Teach Emerg Med*. 2020;5(3):O1–O27. Published 2020 Jul 15. doi:10.21980/J8M93D

### Results and tips for successful implementation

We recommend implementation as part of a mock oral board examination session. Furthermore, we recommend attempting to recreate the ABEM virtual oral boards environment in order to help the learner develop familiarity with what they will see, hear, and need to do on their actual oral boards examination day. The case should be administered by a faculty member who is well prepared and has pre-read the case, stimuli, scoring sheet, and resources.

This case was tested on emergency medicine residents and recent graduates as part of a virtual mock oral board examination day during protected weekly conference time for three institutions on April 4th, 2024. Faculty members, with whom the learners were not familiar, administered the case to the learners (faculty were matched with learners from one of the other collaborating institutions). Formal case scoring sheets were completed by faculty members on each learner. Post-intervention surveys were also administered to all learners and faculty.

Of 49 examinees and 6 faculty examiners, 42 and 4 participated in the assessment, respectively, for an overall 84% response rate. On a Likert scale from 1 (least effective) to 5 (most effective), learners rated the case at a mean 3.9 and standard deviation 0.8, and faculty rated the case at a mean 4.3 and standard deviation 0.6, across the 8 structured interview performance areas. Case difficulty was rated intermediate/advanced by the majority of both learners and faculty. On a Likert scale from 1 (lowest quality) to 5 (highest quality), learners rated the case at a mean 3.9 and standard deviation 0.7, and faculty rated the case at a mean 4.0 and standard deviation 0.0. Learner and faculty participants also provided narrative recommendations which were used to make improvements in the case. Specifically, clarifying the history, adding a point-of-care glucose to the workup, and allowing varied magnesium sulfate dosages within the recommended range for eclampsia was suggested. Outside of medical knowledge aspects of this clinical feedback, there were also comments about the structured interview format being confusing in general.

Overall, the educational content was noted by learners and faculty to be effective, high-quality, and intermediate-to-advanced in difficulty. From this implementation, we discovered that learners need more instruction on magnesium dosing in severe eclampsia, the need for pain medication for sedated patients, and the likelihood of concurrent PRES with eclampsia. Outside of the main medical knowledge take-away lessons, we have also gained insight about lack of familiarity with the structured interview format on the part of both examinees (learners) and faculty examiners (faculty).

Some modifications were made to the case as a result of the implementation. More case evidence for the diagnosis of PRES was added (the patient’s course was changed to reflect confusion for three days even prior to seizure or postictal state). Magnesium sulfate loading doses ranging from 4–6 grams were determined to be acceptable to meet this critical action. Lastly, the critical action of reporting a set of vital signs to the admitting team was changed to explaining the diagnosis and plan to the concerned family.

In addition to case material changes, we suggest that both faculty examiners and learners undergo a formal, guided session during protected conference time to address the nuanced structured interview format before the case is administered. There were a significant number of learners who attempted to manage this as a traditional oral boards case, provided too many items on the differential diagnosis, or made other errors requiring redirection from faculty. There were faculty members who deviated from the structured interview script.

### Pearls

#### Key Learning Objectives and Take-Home Points

- Recognize PRES as a potential diagnosis in pregnant and postpartum women with altered mental status (AMS), headache, and hypertension.
- Identify eclampsia: seizures in a patient with preeclampsia features, even up to four weeks postpartum.
- Learners must clearly articulate a rationale for each clinical decision in structured interviews.

#### History & Physical Pearls

- Always ask about recent pregnancy in reproductive-age females with AMS/seizure.
- Look for evidence of trauma, vision changes, skin changes, edema, and abdominal involvement.
- Thorough neurologic exam is crucial; be alert for seizure activity or postictal states.

#### Workup Essentials

- Head CT to rule out hemorrhage or mass; follow up with MRI if CT is non-diagnostic.
- Urinalysis → check for proteinuria.
- CBC, CMP, coags, peripheral smear → evaluate for hemolysis, elevated liver enzymes, low platelet count (HELLP), and other causes of hypertensive emergency.
- Point of care (POC) glucose → rule out hypoglycemia as a seizure cause.

#### Treatment Priorities

- Airway protection → Intubate if patient is obtunded/seizing/vomiting.
- Magnesium sulfate → 4–6g loading dose for eclampsia seizure prevention.
- Blood pressure control → Use IV antihypertensives (eg, labetalol, hydralazine).
- Analgesia/sedation → Important even when intubated.
- Continuous EEG → Consider ongoing seizure risk.
- Consult OB/GYN → For further maternal/fetal treatment.

#### Diagnosis & Disposition

- Final diagnosis: PRES with eclampsia.
- Disposition: Admit to ICU for neurologic monitoring, blood pressure control, seizure management.
- Transition of care: Communicate clearly with the admitting team and update the family.

## Figures and Tables

**Table:** 

**I. History**		
**1**	○Yes ○No	History of recent pregnancy
**2**	○Yes ○No	Rationale for Prompt 2 #1
**3**	○Yes ○No	Rationale for Prompt 2 #2
**II. Physical Examination**		
**4**	○Yes ○No	Skin exam (petechiae, bruising, etc.)
**5**	○Yes ○No	Neurologic exam (pupils, reflexes, etc.)
**6**	○Yes ○No	Rationale for Prompt 4
**III. Differential Diagnosis**		
**7**	○Yes ○No	Diagnosis #1
**8**	○Yes ○No	Diagnosis #2
**9**	○Yes ○No	Diagnosis #3
**IV. Diagnostic Studies**		
**10**	○Yes ○No	CT Head
**11**	○Yes ○No	CBC, CMP, Coags and/or Type & Screen
**12**	○Yes ○No	POC Glucose
**13**	○Yes ○No	Urinalysis
**14**	○Yes ○No	Rationale for Prompt 7 #1
**15**	○Yes ○No	Rationale for Prompt 7 #2
**V. Treatment and Other Actions**		
**16**	○Yes ○No	Endotracheal intubation
**17**	○Yes ○No	Magnesium sulfate (4-6 g loading dose)
**18**	○Yes ○No	Antihypertensive (eg, labetalol)
**19**	○Yes ○No	Pain control (eg, fentanyl drip)
**20**	○Yes ○No	Rationale for Prompt 9 #1
**21**	○Yes ○No	Rationale for Prompt 9 #2
**VI. Final Diagnosis**		
**22**	○Yes ○No	PRES with Eclampsia
**VII. Disposition**		
**23**	○Yes ○No	Admit to ICU
**24**	○Yes ○No	Rationale for Prompt 12
**VIII. Transitions of Care**		
**25**	○Yes ○No	Report the diagnosis to admitting service
**26**	○Yes ○No	Explain the diagnosis to the family

**Table:** 

Clinical Decision-Making Task Sheet
**CASE PARAMETERS**
This is a 15-minute case. You will interact with two examiners. This is an interview style without role playing; you should simply reply to the questions asked. You may be interrupted to move you through the case; this is not a reflection of your performance.
**PATIENT INFORMATION**
This case is about a 20-year-old female, recently postpartum from uncomplicated pregnancy and vaginal delivery a week ago. She presents via emergency medical services very confused with severe headache, with family reporting three days of worsening symptoms and associated vomiting. On arrival she is hypertensive, in severe distress, clutching her head and wincing, but otherwise the exam is unremarkable.
**VITAL SIGNS**
*BP:* 222/119, *P:* 120, *R:* 24, *T:* 98.7° F (orally), *02Sat:* 97% (room air)
**TASK STATEMENT**
Your tasks are as follows: List pertinent elements of a focused history and physical exam. Develop an appropriate differential and/or provisional diagnosis. Select and interpret appropriate studies. Articulate appropriate patient management including discharge instructions.

**Table:** 

**#1**	**Patient Information Form**
**#2**	**Complete Blood Count**
**#3**	**Comprehensive Metabolic Panel**
**#4**	**Other Laboratory Tests**
**#5**	**Urinalysis**
**#6**	**CT Head (non-contrast)**
**#7**	**MRI Brain (flair sequence)**

**Table:** 

**Patient’s Age:**	**20 years**
**Sex:**	**Female**
**Method of Arrival:**	**Emergency Medical Services**
**Chief Complaint:**	**Altered mental status**
**Person Providing History:**	**Family and Emergency Medical Services (EMS)**

**Table:** 

**Vital Signs:**	
**BP:**	**222/119**
**HR:**	**120**
**RR:**	**24**
**T:**	**98.7**°**F orally**
**O2:**	**97% on room air**

**Table:** 

**WBC**	**7.70 × 10** **^3^** **/μL**
**Hgb**	**10.1 g/dL**
**Hct**	**36.0%**
**Platelets**	**301×10** **^3^** **/μL**

**Table:** 

**Na**	**143 mEq/L**
**K**	**3.5 mEq/L**
**Cl**	**103 mEq/L**
**CO2**	**24 mEq/L**
**BUN**	**18 mg/dL**
**Cr**	**1.01 mg/dL**
**Glucose**	**106 mg/dL**
**AST**	**23 u/L**
**ALT**	**30 u/L**
**Alk Phos**	**35 U/L**

**Table:** 

**INR**	**1.0**
**LDH**	**<0.5 g/L**
**Haptoglobin**	**<140 U/L**
**Peripheral smear**	**Normal**

**Table:** 

**Color**	**Dark**
**Sp Gravity**	**1.030**
**Glucose**	**negative**
**Protein**	**3+**
**Ketones**	**negative**
**Nitrite**	**negative**
**Leuk Est**	**negative**
**WBC**	**0–1**
**RBC**	**0–1**

**Figure:**
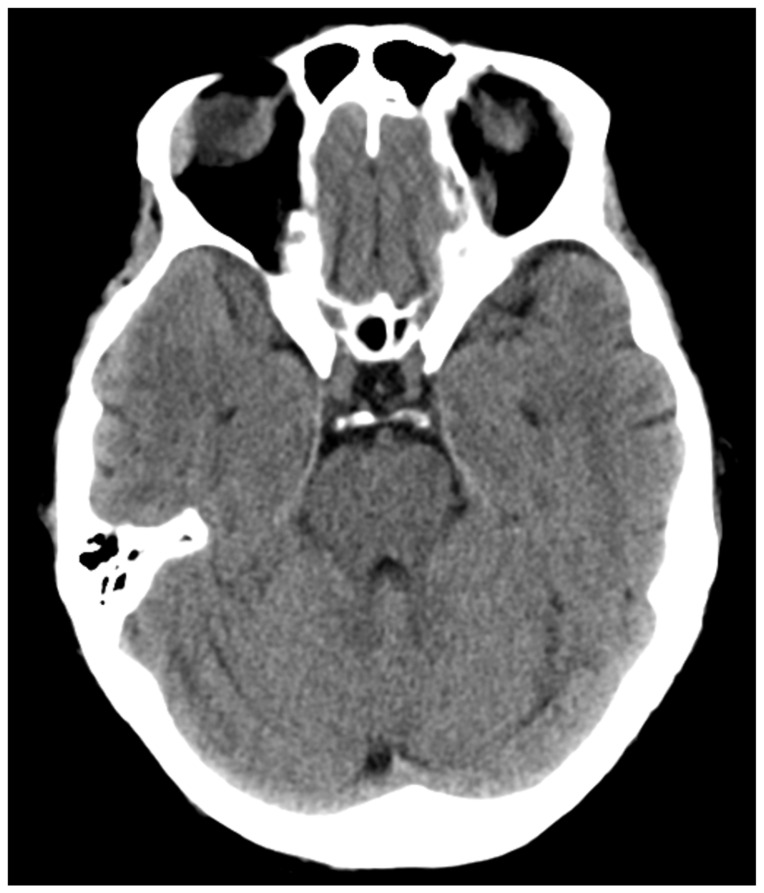


**Figure:**
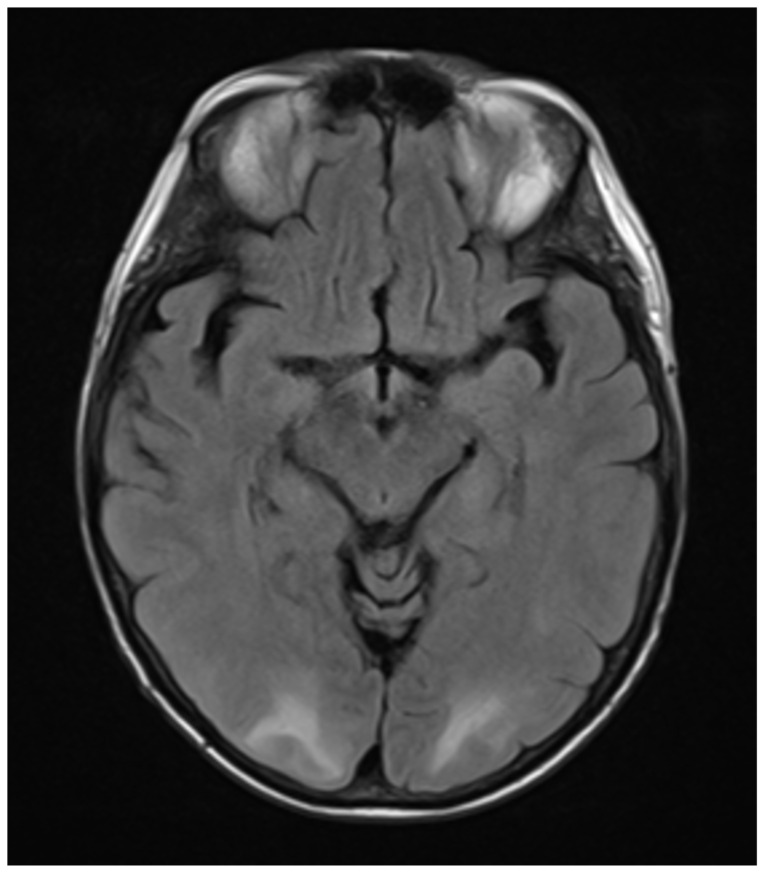


## FOR EXAMINER ONLY

### Standardized Interview Case Summary

**Diagnosis: Posterior Reversible Encephalopathy Syndrome (PRES) and eclampsia**

**Case Summary:** This case is about a 20-year-old female, recently postpartum from uncomplicated pregnancy and vaginal delivery a week ago. She presents via emergency medical services very confused with severe headache, with family reporting three days of worsening symptoms and associated vomiting. On arrival she is hypertensive, in severe distress, clutching her head and wincing, but otherwise the exam is unremarkable.

**Vital Signs:**

Blood Pressure (BP): 222/119
Heart Rate (HR): 120
Respiratory Rate (RR): 24
Temperature (T): 98.7 °F (orally)
Pulse Oximetry (SpO2): 97% (room air)

### Examiner Script

**Case Introduction:** “Hello Doctor, this is a structured interview case. There is no role playing. In response to the questions I will ask, please give me a LIST of information you would gather to come to a final diagnosis. At times, I may interrupt you to move you through the case; this is not a reflection of your performance. You will have 15 minutes to complete the case. Before we begin, do you have any questions?”

“The patient we will be discussing is a 20-year-old female who was brought in by EMS with confusion and headache, with family noting three days of worsening symptoms and associated vomiting today.”

*Provide Learner Stimulus #1*

#### HISTORY

Prompt 1: “Here is the initial information regarding this patient. After you have read it, please give me a list of the additional historical information you would obtain.”

Prompt 2: “You indicated you would ask the patient about X. Why is this important to you?”

Examples: Pregnancy, pregnancy complications, hypertension, seizure disorder, prior headache history, prior trauma, current medications, infectious symptoms.

**ASK THIS PROMPT TWICE ABOUT TWO SEPARATE TOPICS**

*Scoring Guidelines:* Rationale for asking about recent pregnancy is that in childbearing-age females, obstetric complications must be considered and can be life-threatening.

General Guidelines:

- If candidate begins managing the case like a standard case, examiner states, “Remember Doctor, there is no role playing in this case. Please list the additional information you want to obtain.”
- If candidate does not offer a complete list of historical information, examiner should pause long enough to allow them to list additional items, before asking “why” questions.
- If candidate mentions “past medical history,” or “social history,” examiner clarifies by asking, “What specifically do you want to know about PMH/social history?”

#### PHYSICAL EXAMINATION

“You are provided with the following additional historical information:

- Family notes the patient has been increasingly confused for three days while simultaneously developing the headache. She has vomited a few times as well in the past day.
- The family denies that the patient has complained of any recent fever, chills, numbness, tingling, weakness. They have been watching her closely and deny loss of consciousness.
- Family notes the patient had uncomplicated vaginal delivery of her first baby one week ago and was doing well until the past three days. The patient had no known pregnancy complications and was followed by OB/GYN throughout her pregnancy.”

Prompt 3: “Based on what you now know, please give me a list of specific physical examination findings you would be looking for.”

Prompt 4: “Doctor, you examined X during the physical exam. Please explain how that would help you.”

Examples: skin exam, extremity exam, neurologic examination, visual exam, abdominal exam (such as RUQ tenderness)

*Scoring Guidelines:* Rationale for checking skin exam: evaluating for petechial rash (meningitis), bruising (trauma, disseminated intravascular coagulation), jaundice. Rationale for checking neurologic exam: it is essential to evaluate for continual seizure-like activity, focal deficits, Glasgow coma scale (GCS) level, etc. Rationale for checking vision exam: evaluation of pupillary reflexes as well as vision changes/loss to assess for occipital lobe involvement. Rationale for checking abdominal exam: evaluating location of tenderness as well as presence/absence of rebound/guarding can evaluate for potential liver involvement in the RUQ or retained products if in the lower abdomen.

#### DIFFERENTIAL DIAGNOSIS

“You are provided with the following physical exam findings:

- Patient is confused, not following commands, in severe distress, clutching her head. Head appears atraumatic.
- Heart tachycardic. No murmurs, rubs or gallops. Lungs clear to auscultation bilaterally with good air movement, abdomen nontender.
- Skin/mucosa exam reveals mildly pale conjunctiva, no jaundice, no skin/mucosal lesions.
- Extremity exam reveals 1+ pitting edema to bilateral lower extremities with no tenderness.
- A truncated neurologic examination is initially normal, including equally round and reactive pupils, normal reflexes.
- However, the patient has a generalized tonic-clonic seizure toward the end of the exam, lasting 5 minutes, after which time she is obtunded, vomiting repeatedly, and having difficulty breathing.”

Prompt 5: “Based on what you now know, what are the top three items on your differential diagnosis based on the most likely conditions?” (If more than three conditions are mentioned, say, “OK thank you. Please give me your three, and only three, most likely diagnoses.”)

Appropriate differential diagnoses include:

- PRES (posterior reversible encephalopathy syndrome)
- Eclampsia
- Intracranial hemorrhage
- Hypertensive emergency
- HELLP (hemolysis, elevated liver enzymes, low platelets)
- Venous, air, or amniotic fluid embolism
- Intracranial hypotension and cerebral spinal fluid (CSF) leak from epidural
- Stroke
- Encephalitis/meningitis

*Scoring Guidelines:* If the candidate lists “pregnancy complication” or “hypertension” as an item on differential, the examiner should ask them to be more specific.

#### DIAGNOSTIC STUDIES

Prompt 6: “Based on what you know and your working differential diagnosis, what, if any, diagnostic studies would you order?”

Prompt 7: “Doctor, you ordered X. Why X?”

Examples: head computed tomography (CT), urinalysis (UA), type and screen, complete blood count (CBC), comprehensive metabolic panel (CMP), POC glucose

**ASK THIS PROMPT TWICE ABOUT TWO SEPARATE TOPICS**

*Scoring Guidelines:* Rationale for ordering Head CT is to assess for intracranial abnormality such as PRES, mass, or hemorrhage. Rationale for ordering glucose level is to evaluate hypoglycemia as a possible reversible cause of seizure/altered mental status. Rationale for ordering UA is to assess for proteinuria, urinary infection.

*After the candidate responds*,
*PROVIDE STIMULUS 2, 3, 4, 5, 6, and 7, depending on what the learner ordered.*

#### TREATMENT AND OTHER ACTIONS

Prompt 8: “Based on what you now know, what treatments, if any, would you order and/or what actions, if any, would you perform?”

Prompt 9: “Doctor, you ordered X. Why X?”

Examples: endotracheal intubation, magnesium sulfate (bolus and drip for seizures), antihypertensive medication, pain medication, continuous EEG monitoring

**ASK THIS PROMPT TWICE ABOUT TWO SEPARATE TOPICS**

*Scoring Guidelines:* Rationale for rapid sequence intubation is that the patient is obtunded, vomiting, likely had two seizures. Assume unable to protect her airway. The rationale for pain medication is that this is important even after the patient is sedated and intubated. Not all sedative drips have analgesic properties, and this patient had a severe headache. Appropriate options include propofol, versed, or fentanyl. Ketamine is not initially appropriate given the patient’s tachycardia and severe hypertension. Continuous EEG can be considered since ongoing seizures are a risk.

#### FINAL DIAGNOSIS

Prompt 10: “Based on everything you know about this case, what is your final diagnosis?”

- PRES with Eclampsia

*Scoring Guidelines:* Verbalizing “PRES with eclampsia” meets the critical action. If the candidate mentions something vague such as “obstetric complication” or “new onset seizure disorder: examiner asks, “can you be more specific about the diagnosis?”

#### DISPOSITION

Prompt 11: “Based on what you know, what should be the disposition of this patient?”

Prompt 12: “Why would you (admit/discharge) this patient?”

*Scoring Guidelines:* Verbalizing disposition to the ICU and explaining why it is necessary meets the critical action. This patient has been intubated and has life-threatening postpartum complications, requires frequent neurologic checks, titratable drips (sedative, antihypertensive), continuous EEG, etc.

#### TRANSITION OF CARE

Prompt 13: “What specific actions would you take at the time of (admission/discharge)?”

*Scoring Guidelines:* Verbalizing final actions such as calling the critical care specialist, signing out the case, and explaining the diagnosis to the family meets the critical action.

*Thank you, Doctor. That concludes this case.*

*Please tear up your notes.*

## ORAL BOARDS ASSESSMENT

*Posterior Reversible Encephalopathy Syndrome and Eclampsia*

Learner: _________________________________________

### Stimulus Inventory

#### Stimulus #1

##### Structured Interview: Patient Information

**General Appearance/History of Present Illness:**

20-year-old female brought in by EMS very confused, in severe distress due to pain, and vomiting. Family notes she has had three days of worsening headache and confusion, and she vomited a few times in the past day. EMS states they were called by family because the patient’s headache and confusion worsened this morning. Family is in the department and is very worried, asking what is wrong with the patient.

## DEBRIEFING AND EVALUATION PEARLS

### Posterior Reversible Encephalopathy Syndrome and Eclampsia

**Key Learning Objectives and Take-Home Points:**

- Recognize PRES as a potential diagnosis in pregnant and postpartum women with altered mental status (AMS), headache, and hypertension.
- Identify eclampsia: seizures in a patient with preeclampsia features, even up to four weeks postpartum.
- Learners must clearly articulate a rationale for each clinical decision in structured interviews.

**History & Physical Pearls:**

- Always ask about recent pregnancy in reproductive-age females with AMS/seizure.
- Look for evidence of trauma, vision changes, skin changes, edema, and abdominal involvement.
- Thorough neurologic exam is crucial; be alert for seizure activity or postictal states.

**Workup Essentials:**

- Head CT to rule out hemorrhage or mass; follow up with MRI if CT is non-diagnostic.
- Urinalysis → check for proteinuria.
- CBC, CMP, coags, peripheral smear → evaluate for hemolysis, elevated liver enzymes, low platelet count (HELLP), and other causes of hypertensive emergency.
- Point of care (POC) glucose → rule out hypoglycemia as a seizure cause.

**Treatment Priorities:**

- Airway protection → Intubate if patient is obtunded/seizing/vomiting.
- Magnesium sulfate → 4–6g loading dose for eclampsia seizure prevention.
- Blood pressure control → Use IV antihypertensives (eg, labetalol, hydralazine).
- Analgesia/sedation → Important even when intubated.
- Continuous EEG → Consider ongoing seizure risk.
- Consult OB/GYN → For further maternal/fetal treatment.

**Diagnosis & Disposition:**

- Final diagnosis: PRES with eclampsia.
- Disposition: Admit to ICU for neurologic monitoring, blood pressure control, seizure management.
- Transition of care: Communicate clearly with the admitting team and update the family.
